# Optical modulation of microfibers and application to ultrafast fiber lasers

**DOI:** 10.1039/c8ra00740c

**Published:** 2018-03-01

**Authors:** Ruwei Zhao, Guoru Li, Baitao Zhang, Jingliang He

**Affiliations:** State Key Laboratory of Crystal Materials, Shandong University Jinan 250100 China jlhe@sdu.edu.cn

## Abstract

Microfibers with different waist diameters were prepared successfully by a flame-brushing technique. Their saturable absorption properties were investigated. The non-saturable loss and modulation depth both decreased with the increase of the diameter. According to the mode distribution of microfibers with a waist diameter of 25 μm, it could be supposed that the evanescent field effect may be useful for microfibers being saturable absorbers (SAs). Based on the 25 μm-diameter microfiber, an all-fiber-structure mode-locked fiber laser was achieved successfully with long term stability in the span of a week. The results indicated that microfibers with suitable diameters were excellent SAs. To the best of our knowledge, this is first report on the usage of microfibers as a SA for building ultrafast fiber lasers.

## Abstract 2

Microfibers with suitable diameters were used as saturable absorbers, realizing an all-fiber-structure mode-locked fiber laser.

## Graphical abstract

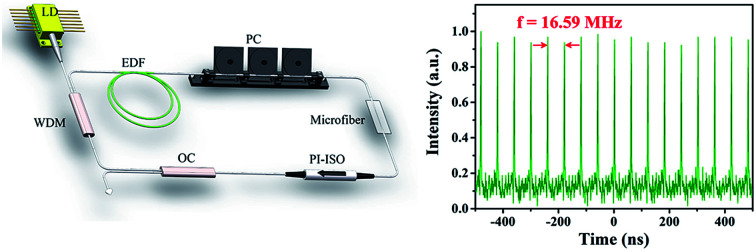

## Introduction

Integrating fiber optics and nano-technology, optical microfibers have attracted growing interest for varied applications in the fields of communication, sensors, nonlinear optics and quantum optics.^1,2^ In the past few decades, microfibers have been manufactured and applied in a wide range of fiber optic technology because of their flexibility and extraordinary optical and mechanical properties.^3,4^ Early in 1989, an 8.5 μm-diameter microfiber was used to realize the ring resonator and high *Q* of 27 000 at communication band.^5^ Except the ring resonator, other types of resonators were demonstrated with microfibers, for instance, the multicoil and knot cavity.^6,7^ For the properties of smooth surface, low loss and strong evanescent filed, microfiber are also of great importance in optical device engineering, such as compact couplers, Mach–Zehnder interferometer, sensors and optical filters.^8,9^ Actually, microfiber could be used not only in the passive resonator, but to construct micro-fiber-laser. In 2006, Jiang *et al.* demonstrated a 1.5 μm microfiber laser formed by tightening a tapered Er:Yb-doped fiber into a knot successfully.^10^ Besides incorporating active materials into the fiber, the strong evanescent field of microfiber offers another approach to dope the gain materials outside of tapered region. Based on this method, Jiang *et al.* realized a knot dye laser providing a possibility for application of microfibers to optofluidic system.^11^ In addition, supercontinuum generation using microfibers was also investigated.^12^ The microfiber provide higher confinement than standard single mode fibers (SMFs) for the strong optical nonlinear effect of tapered fiber,^13^ which provided an exciting opportunity for the application in nonlinear optics.

Optical modulation is one of crucial operations in photonics.^14^ Recently, two-dimensional materials (such as graphene, transition metal dichalcogenides, and black phosphorous) have been extensively studied in optical technology. By incorporating the materials onto microfiber *via* the strong evanescent filed, mode-locked fiber laser can be easily achieved, which provide the application in optical modulator of microfiber.^15–17^ However, whether the microfiber itself is competent as SA to realize ultrafast laser has not been reported yet.

In this work, the nonlinear optical properties of microfibers with different diameters have been experimentally studied. The microfibers were fabricated using flame-brushing technique. With the I-scan measurement, the saturable absorption property of microfiber was characterized. The experimental results displayed that the non-saturable loss and modulation depth both decrease with the increase of diameter of microfibers. What's more, based on a 25 μm-diameter microfiber, we have successfully realized an all-fiber-structure dual-wavelength mode-locked laser with the maximum average output power of 19 mW. The spectrum with the peaks at 1593.9 and 1595.3 nm had full width half maximum (FWHM) of 0.64 and 0.72 nm, respectively. The pulse repetition rate third-order harmonic mode-locked fiber laser was also achieved, just by increasing the pump power. Besides, the pulsed laser long-time stability in a span of a week was studied. The results proved microfiber with suitable diameter was an excellent saturable absorber. To the best of our knowledge, it is the first time to investigate the optical modulation of microfibers and application in the ultrafast fiber laser.

## Preparation and characterization of microfibers

**Fig. 1:**
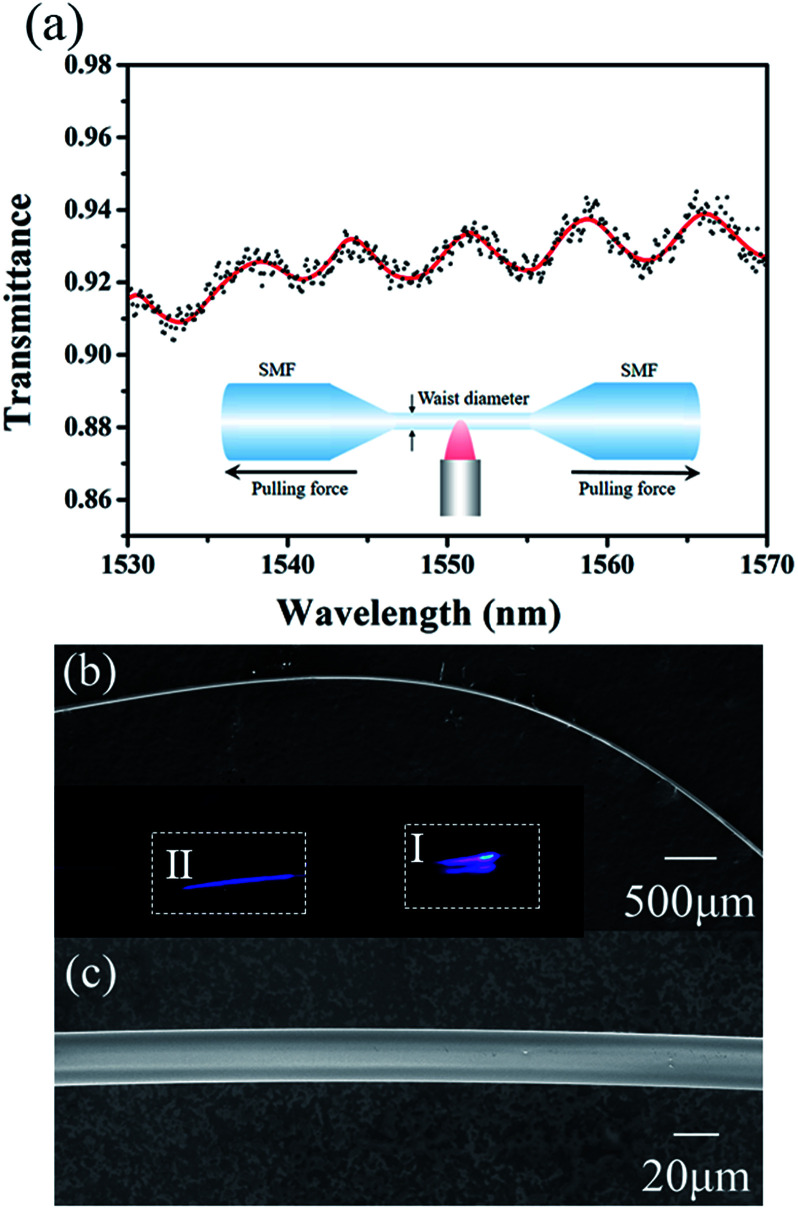
(a) The transmission spectra of 25 μm-waist-diameter microfiber. Inset is the fabrication and structure of microfiber. SEM image of microfibers with waist diameter of 25 μm in (b) 500 μm scale bar and (c) 20 μm scale bar. The inset is evanescent field of microfiber observed by a visible 455 nm light.

Compared to other manufacture of microfiber such as chemical growth and nano-imprint, flame-brushing technique yield microfibers with low surface roughness, large length and excellent diameter uniformity.^1,18^ Here we fabricated the microfibers using flame-brushing technique as shown in the inset of Fig. 1(a). The bare standard SMFs were stretched in virtue of a flame. By adjusting the pulling distance, microfibers with the taper waist diameter of 25, 38, 55, 65, and 78 μm were achieved. The transmission spectra of 25 μm-waist-diameter microfiber is shown in Fig. 1. Due to the scope limit of the pump source, the transmission spectra was measured in the range of 1530–1570 nm. The result shows that the microfiber has a weak filtering effect, which may be useful for the generation of dual-wavelength laser. The scanning electron microscopy (SEM) was usually used to study the morphologies of sample. Here, Fig. 1(b) and (c) showed SEM image of microfiber with the waist diameter of 25 μm using different scale bars, which proved high evenness degree and smooth surface of prepared microfibers. With a visible 455 nm laser guided through the 25 μm-diameter microfiber, it was clearly seen the evanescent field from scattered light as displayed in inset of Fig. 1. The microfiber was fixed on a slide glass by the adiabatic tape. The refractive index of microfiber and the slide glass is 1.46 and 1.5, respectively. For comparison, the evanescent field of bare SMF was also shown in Section II. Obviously, the evanescent field of tapered fiber as shown in Section I was much higher than bare SMF. The mirror image in Section I was caused by the reflection of slide glass.

**Fig. 2:**
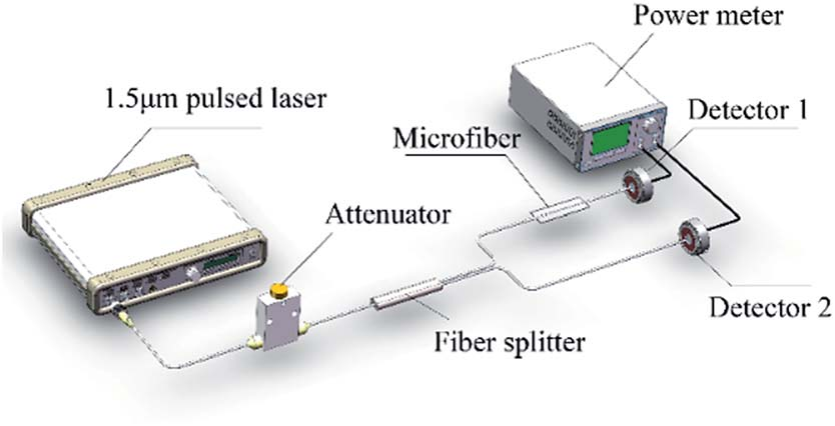
Experimental setup for nonlinear absorption measurement of microfibers.

**Fig. 3:**
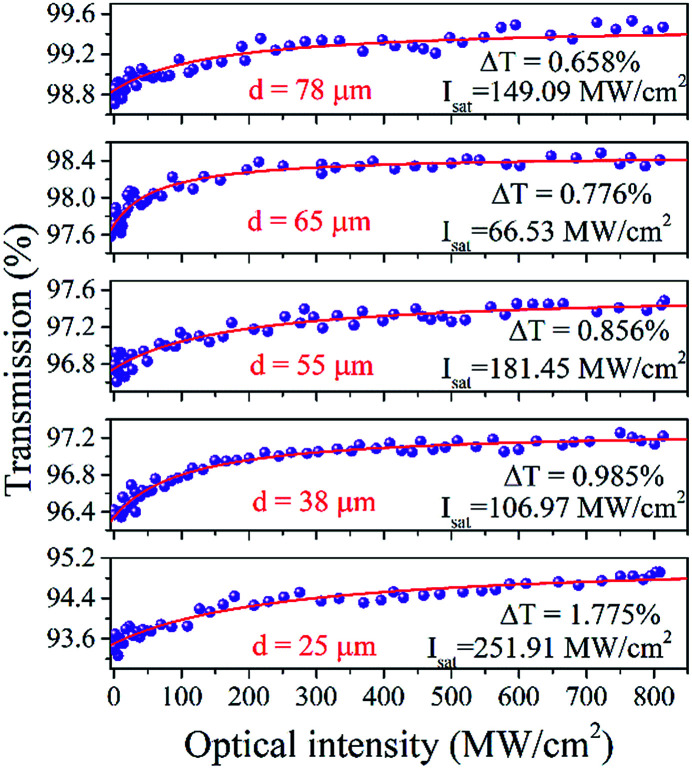
Transmittance of microfibers as a function of pulse intensity with different waist diameters.

Open-aperture Z-scan and I-scan are the common techniques for the test of nonlinear optical absorption of materials. Several advantages exit in performing I-scan over the Z-scan technique, such as fully fiber-integrated setup or thin-enough sample.^19^ We measured the nonlinear optical absorption of microfiber by I-scan technique firstly, as described in Fig. 2. The probe laser is a self-constructed mode-locked fiber laser centered at 1557 nm, with the pulse width and repetition rate of 750 fs and 15.6 MHz, respectively. The laser with maximum output power of 50 mW can be adjust by an attenuator. After that, the pulse was split equally with a fiber coupler, in which one branch performed as a reference beam and the other was connected with the prepared microfiber. By comparing the pulse intensities of the two branches, the transmissions *versus* the pulse intensity were obtained. The results are as described in Fig. 3. The experimental data were fitted according to the equation:^20^1
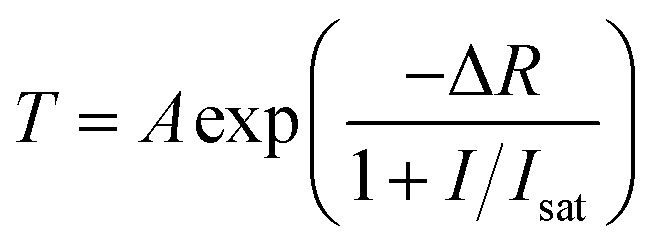
where *A* is a normalization constant, *T* is the transmission of microfiber, *I* is the incident intensity, Δ*R* and *I*_sat_ are the modulation depth and saturation intensity. The related goodness-of-fit (*R*^2^) is 90.53%, 91.52%, 91.29%, 96.67%, 94.71%, respectively. Details were shown in Table 1. The greatest contribution to the transmission loss of microfiber comes from the surface imperfections, cracks, and impurities connected with the microfiber surface. Obviously, the non-saturable loss increases with the decreasing of the waist diameter under the same manufacturing technique. The trend is in good agreement with precious reports on the propagation loss of microfibers.^21^ To our amusement, the modulation depth is inversely proportional to waist diameter of microfiber, which is an interesting conclusion on the properties of microfibers. However, the saturation intensity did not significantly laws with the change of the waist diameter of microfibers. We surmise the reason may be caused by the measurement error and fitting error.

**Table 1:** Detail results of nonlinear properties of microfibers with different waist diameter

Waist diameter (μm)	Non-saturable losses	Modulation depth	Saturation intensity (MW cm^−2^)
78	0.59%	0.66%	149.09
65	1.55%	0.78%	66.53
55	2.56%	0.86%	181.45
38	3.78%	0.98%	106.79
25	5.11%	1.78%	251.91

**Fig. 4:**
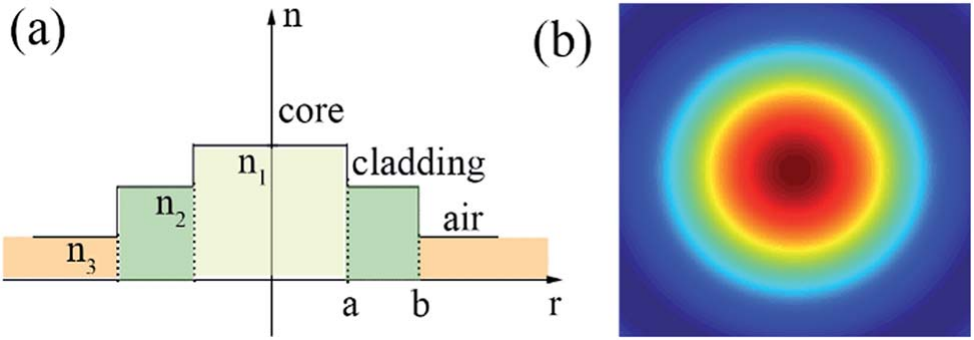
(a) Index profile of the tapered fiber. (b) The radial field distribution of HE_11_ modes of microfiber with the diameter of 25 μm.

Considering the enough large waist diameter (25 μm) of microfiber compared to the light wavelength (1.55 μm), the three layered tapered fiber model was adopted, as shown in Fig. 4(a). Combined Maxwell equations with boundary condition, the eigenvalue equations of HE_mn_ modes could be described as following formula:^22,23^2
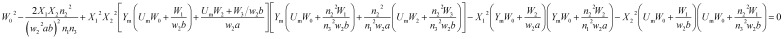
where,
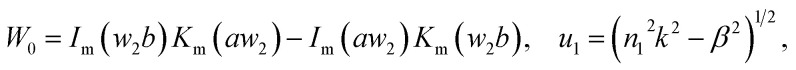

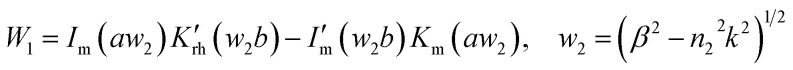

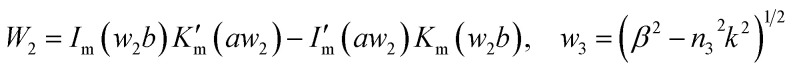

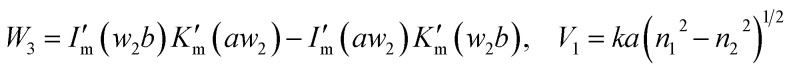

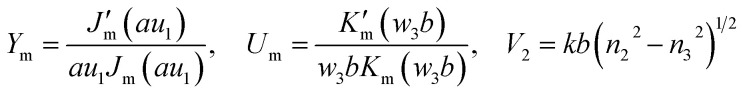

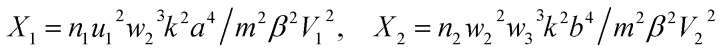
*J*_m_ is the Bessel function of the first kind, *I*_m_ and *K*_m_ are the modified Bessel function of the first kind and second kind, respectively. *k*_0_ = *2*π*/λ*, *a*, *b*, *n*_1_ and *n*_2_ are radius and refractive indices of core and the cladding of microfiber, *n*_3_ is the refractive index in air, *β* and *λ* are propagation constant and the wavelength of light, respectively. According to the optical waveguide theory, the radial field can be described as follows:3
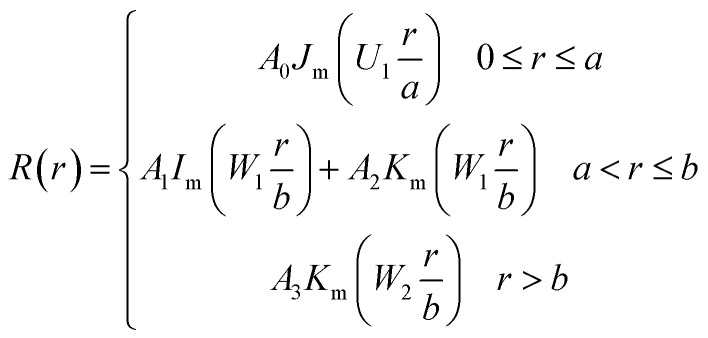


By solving eqn (3), Fig. 4(b) shows the power distribution of HE_11_ mode of the tapered fiber with the diameters of 25 μm. It is clear that a part of energy guided outside of the cladding of microfibers as evanescent waves. So we guess the reason for microfibers being a SA may be the evanescent field effect. Further investigation will be necessary.

## Experimental setup

**Fig. 5:**
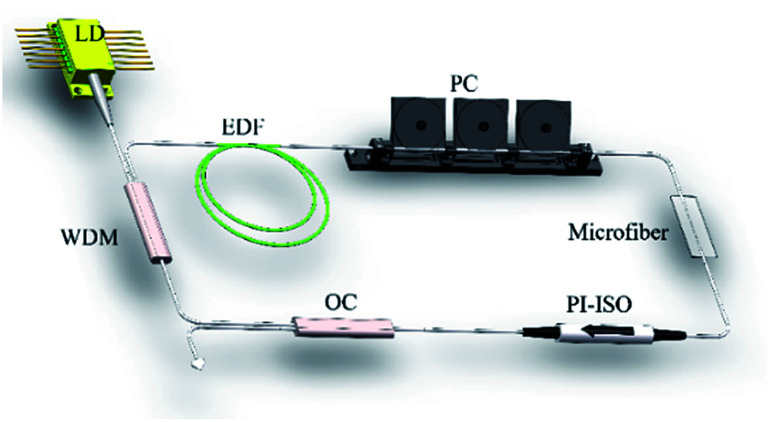
Experimental setup of the mode-locked fiber laser based on microfibers.

To test the saturable absorption ability of microfibers, we proposed a compact fully fiberized cavity, as schematically shown in Fig. 5. A 976 nm laser diode (LD) with maximum power of 500 mW was coupled into the gain medium *via* a 976/1550 wavelength division multiplexer (WDM). 0.9 m-highly-doped erbium fiber laser (EDF, LEKKI Er 110-4/125) was used with dispersion parameter of −12 ps nm^−1^ km^−1^. A polarization-independent isolator (PI-ISO) was required to ensure unidirectional operation of the fiber laser. The polarization states of propagation light were rotated through a polarization controller (PC). The laser was coupled out through a 10% optical coupler (OC). The rest fiber of ring cavity, including the pigtails of microfiber and various components, were all SMF with dispersion parameter of 18 ps nm^−1^ km^−1^. The net dispersion was calculated to be about −0.25 ps^2^ with the total cavity length of 12.4 m. The laser performance is monitored by an optical power meter (THORLABS, S148C), a 1 GHz digital oscilloscope (Tektronix DPO 7104) coupled with a 1 GHz photodetector, an optical spectrum analyzer (Yokogawa AQ6370C) and a 3 GHz RF spectrum analyzer (Agilent N900A).

## Results and discussions

**Fig. 6:**
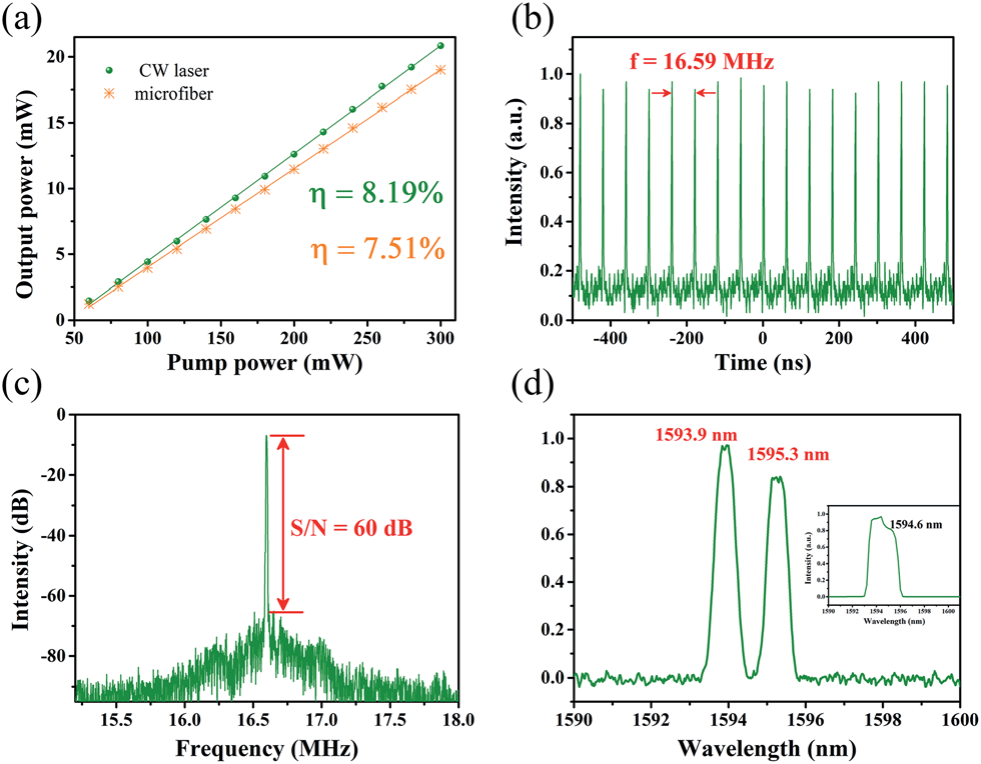
Testing results of the microfi ber mode-locked fiber laser. (a) Output power as a function of pump power with (yellow) and without (green) microfiber. (b) Typical pulse train. (c) Radio frequency spectrum. (d) Spectrum, inset: laser spectrum without microfiber.

First, to distinguish the proposed mode-locked fiber laser from the nonlinear polarization rotation scheme,^24^ the polarization property of microfiber using a polarized light with the wavelength of 1550 nm was test. By monitoring the change of output power through 25 μm-waist-diameter microfiber, obviously, the output power was almost no change. The result indicates that microfiber SA is polarization-independent. Then the ring cavity without inserting microfiber was executed. Only continuous wave (CW) could be observed when adjusted PC or increased pump power. The pump threshold of CW operation was 17 mW with slope efficiency of 8.19%, as displayed in Fig. 6(a). After microfiber with waist diameter and taper length of 25 μm and 15 mm incorporating, the threshold for CW laser increased up to 41 mW with the slope efficiency of 7.51%. By slight adjusting the PC, the mode-locked fiber laser emerged when pump power further increased to 120 mW. For better performance of mode-locked fiber laser, the results were recorded when the pump power increased to 210 mW. A typical pulse train is depicted in Fig. 6(b). Fundamental repetition rate was 16.59 MHz, which was determined by the cavity length of 12.4 m and verified the mode-locking state. The corresponding radio frequency (RF) spectrum was shown in Fig. 6(c), with resolution bandwidth (RBW) of 20 kHz. The signal-to-noise (S/N) ratio was larger than 60 dB, indicating good stability of mode-locked fiber laser based on microfiber. The results proved microfibers can be a good candidate as SA.

The emission spectrum is shown in Fig. 6(d). It can be clearly observed dual-wavelength mode-locked fiber laser with the peaks of 1593.9 and 1595.3 nm was generated. The two peaks were both Gaussian-like profile with the FWHM of 0.64 and 0.72 nm, respectively. The spectrum showed clearly there was no Kelly sidebands, which may be caused by the spectral filtering effect. In this experiment, we didn't measure the real pulse width for the lack of suitable autocorrelator. However, according the soliton theory, the theoretical limit pulse duration could be estimated. The two peaks of spectrum with different FWHMs implied two separate pulse trains. The pulse duration of the two pulses was calculated to be 4.18 ps and 3.72 ps, respectively. For comparison, the laser spectrum of CW operation was also recorded, as shown in the inset of Fig. 6(d). No obvious dual-spectrum was observed. So we can reasonably infer that the microfiber contributed to generation of dual-wavelength laser, which can be explained as follows: microfiber in the cavity not only worked as a SA, but a Mach–Zehnder interferometer (MZI). The MZI as fiber comb filter is an excellent method for generate multi-wavelength laser.^25^

**Fig. 7:**
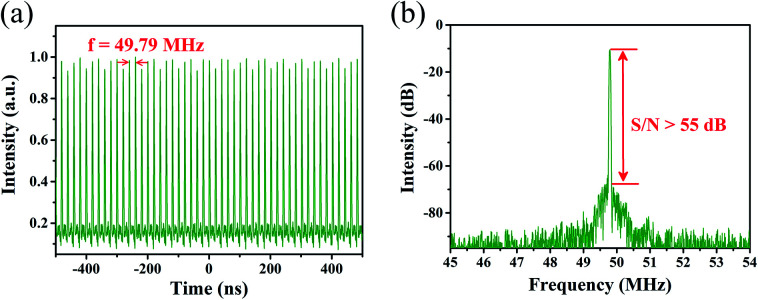
Testing results of harmonic mode-locked fiber laser based on microfiber. (a) Typical pulse train. (b) Radio frequency spectrum.

Continue to raise pump power to 220 mW, a new type of stable pulse train emerged, as shown in Fig. 7(a). The repetition rate was 49.79 MHz, correspondent to third harmonic of fundamental repetition rate. The change of repetition rate only depends on the pump power. There is no need to rotate the PC. The RF spectrum was depicted in Fig. 7(b), with the RBW of 4.7 kHz. The S/N ratio was higher than 55 dB, indicating the harmonic mode-locked fiber laser was also in quite stable state. Adding the pump power to 250 mW, the stable mode-locking fiber laser was collapsed suddenly. However, reducing the pump power again, the harmonic and fundamental mode-locking pulses can be both reconstructed. As a result, the microfiber was confirmed owning a fairly high damage threshold.

**Fig. 8:**
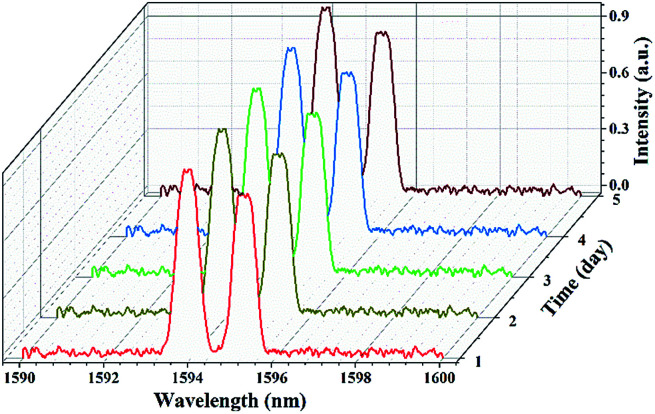
The measured spectra of fundamental mode-locked fiber laser at 1 day interval within a span of a week.

Besides, the repeat scans of spectra measured within a span of a week were also recorded, as illustrated in Fig. 8. It can be verified that the mode-locked fiber laser based on microfiber with suitable diameter has a fairly long-term stability. The results proved the unique superiority of microfiber on the application to ultrafast fiber laser. We also demonstrated the same cavity with other prepared microfibers with different waist diameter. Except the 25 μm-diameter-microfiber, there is no stable mode-locking fiber laser, regardless of how to rotate PC or change pump power. The result displayed the waist diameter of microfiber is a main factor for the application as a SA. However, microfibers with smaller-diameters have not been fabricated due to the restriction of preparation. It is still hard to make deterministic conclusion on the selection of microfiber diameter as a SA. Specific origins of saturable absorption of microfibers are not so clear. Further detailed investigation will be essential.

## Conclusions

In conclusion, a series of high-quality microfibers with different waist diameter was prepared by using flame-brushing technique. The saturable absorption property of microfibers were studied with I-scan measurement. The non-saturable loss and modulation depth both decreased with the increase of diameter of microfibers. Based on the microfiber with suitable diameter, an all-fiber-structure dual-wavelength mode-locked fiber laser was achieved. To our best of knowledge, it is the first time to investigate the optical modulation of microfiber and application to the ultrafast fiber laser. The long-term stability and repetition rate third harmonic mode-locked laser have fully testified microfiber could be a kind of excellent SAs for generating pulsed lasers.

## Conflicts of interest

There are no conflicts to declare.

## Supplementary Material
